# Improving Femoral Tunnel Placement in Canine Cranial Cruciate Ligament Reconstruction Using a Novel Adjustable Aiming Device: Evaluation Through Simulated Surgical Trials

**DOI:** 10.3390/ani16213345

**Published:** 2026-10-23

**Authors:** Tsai-Yu Lee, Cheng-Chung Lin, Shih-Yuan Yang, Yu-Chih Wang, Ching-Ho Wu

**Affiliations:** 1 Department of Surgery, National Taiwan University Veterinary Hospital, Taipei 10617, Taiwan; eloiselee128@gmail.com; 2 Department of Electrical Engineering, Fu Jen Catholic University, New Taipei 242062, Taiwan; 129787@mail.fju.edu.tw; 3 Institute of Veterinary Clinical Science, National Taiwan University, Taipei 10617, Taiwan; r12643015@ntu.edu.tw (S.-Y.Y.); r11643004@ntu.edu.tw (Y.-C.W.)

**Keywords:** cranial cruciate ligament, intra-articular reconstruction, aiming device, femoral tunnel placement, patient-specific instrumentation

## Abstract

Accurate femoral tunnel placement in canine cranial cruciate ligament (CrCL) reconstruction is challenging because of small joint anatomy and limited dedicated instrumentation. This study evaluated an adjustable femoral tunnel aiming device combined with patient-specific preoperative planning. Thirteen surgeons (six experienced and seven novices) performed simulated tunnel positioning using the device and a freehand technique. Outcomes included aperture positional error, measured as Euclidean distance (ED), angular deviation (AD), positioning time, and intra- and intersurgeon variability. Device-guided positioning reduced EDs (4.1–4.7 mm vs. 5.1–6.2 mm; *p* = 0.0275) and ADs (6.4–8.2° vs. 10.7–12.6°; *p* = 0.0038). Neither surgeon experience nor its interaction with technique significantly affected EDs or ADs. The device significantly reduced intrasurgeon variability in both outcomes and intersurgeon ADs variability among experienced surgeons. However, systematic distal bias (3.1–3.4 mm; *p* < 0.001) was observed with device-guided positioning. These findings support improved positioning accuracy and consistency with the device in simulated canine CrCL reconstruction. Further device refinement and hands-on practice may help address systematic deviations, while additional cadaveric and clinical validation is required before clinical application.

## Simple Summary

Rupture of the cranial cruciate ligament that stabilizes the stifle is a significant cause of lameness in dogs. Reconstructing this ligament requires accurate placement of a tunnel in the thigh bone, which is difficult because few surgical tools are designed specifically for this procedure. We developed an adjustable aiming device to help surgeons position this tunnel. Thirteen surgeons with different levels of experience tested the device and a conventional freehand approach in simulated procedures. The device improved the accuracy of tunnel position and direction and made repeated placements by individual surgeons more consistent. However, placements with the device tended to deviate from the planned position toward the stifle joint. These findings suggest that the device could help veterinarians perform more accurate and consistent ligament reconstruction in dogs.

## 1. Introduction

Cranial cruciate ligament (CrCL) rupture is among the most common orthopedic conditions in dogs and is a major cause of stifle instability and progressive osteoarthritis [1]. In human medicine, anatomic reconstruction of the anterior cruciate ligament (ACL), in which a graft is positioned within bone tunnels at the native femoral and tibial footprints, is widely regarded as the gold standard for treating ACL deficiency and restoring knee stability [2,3]. Compared with nonanatomic reconstruction techniques, anatomic reconstruction has been associated with improved knee kinematics [4], a reduced pivot shift phenomenon [5], reduced graft failure rates [5], and a lower long-term osteoarthritis incidence [6]. However, in veterinary orthopedics, early intracapsular reconstruction (ICR) techniques for CrCL rupture in dogs have demonstrated limited clinical success [7,8,9]. These procedures were generally performed using nonanatomic techniques, which may have contributed to the suboptimal outcomes reported in early canine ICR studies [7,9], in addition to potential anatomical differences between the canine stifle and human knee. More recently, in vivo studies have explored anatomic CrCL reconstruction using deep digital flexor tendon grafts placed within bone tunnels, demonstrating the potential to improve joint stability and restore more physiologic joint kinetics [10,11]. To further investigate the effects of anatomic ICR on postoperative stifle biomechanics in vivo or ex vivo, a surgical technique that allows accurate and reproducible execution of the procedure is needed, particularly with respect to precise tunnel placement for achieving anatomic graft positioning.

Improper placement of the femoral tunnel is among the most common technical errors in human ACL reconstruction and is often associated with suboptimal clinical outcomes, including graft failure and persistent knee instability [12]. Graft malposition may also lead to a restricted range of motion [13], meniscal injury [14], and cartilage degeneration [14]. A biomechanical study further revealed that variations in the femoral tunnel position influence graft length to a significantly greater extent than changes at the tibial insertion [15]. To improve the accuracy of femoral tunnel placement, several surgical techniques have been developed for human ACL reconstruction. These approaches include the transtibial (TT), anteromedial (AM) portal, and the outside-in (OI) technique for femoral tunnel creation [16]. Although the TT technique is technically straightforward, it often results in nonanatomic femoral tunnel positioning, which may compromise rotational stability and limit its ability to fully address the pivot shift phenomenon [16,17]. The AM technique allows for more anatomical tunnel placement but has been associated with risks, including shorter femoral tunnels, posterior wall blowout, damage to the medial femoral condyle, and increased graft stress [18,19,20]. In contrast, the OI technique enables longer, more anatomic femoral tunnels and may reduce the risk of posterior wall blowout and cartilage damage [16]. It also allows accurate tunnel placement without complete visualization of the ligament footprint, which may benefit both open and arthroscopic procedures in dogs.

Accurate localization of the ligament insertion sites is essential for anatomic ICR. However, identifying reliable reference landmarks for tunnel placement in the canine stifle remains challenging. Unlike the human ACL, where several anatomical landmarks have been described to guide footprint identification [21,22,23,24,25,26,27,28], the canine CrCL lacks clearly defined intra-articular reference landmarks such as the lateral intercondylar ridge [29], and its footprint exhibits considerable anatomical variability [30]. In addition, the relatively small size of canine stifles magnifies the effects of angular deviations during tunnel drilling and limits intraoperative visualization of the femoral footprint, particularly given the limited availability of small-diameter arthroscopes in many clinical scenarios. These factors increase the risk of tunnel misplacement when freehand drilling techniques are used.

To the best of our knowledge, only a limited number of aiming devices have been proposed to assist bone tunnel positioning in canine CrCL reconstruction. Winkels et al. developed an adjustable tibial aiming device guided by proximal tibial depth measurements obtained from lateral radiographs, which demonstrated accurate tunnel placement in a cadaveric study under endoscopic guidance [31]. Bolia et al. later introduced a femoral aiming device on the basis of previously reported radiographic locations of the CrCL femoral footprint [32]. However, this device uses fixed aiming angles, and the method for guide positioning was not fully described, which may limit reproducibility across surgical settings. In addition, the reliance on arthroscopic assistance may restrict clinical applicability in settings where such equipment is not readily available. Together, these limitations highlight the need to develop a guidance device that facilitates adjustable and reproducible femoral tunnel placement in canine CrCL reconstruction.

Therefore, the objectives of this study were to develop a femoral bone tunnel aiming device integrated with a customized preoperative planning workflow for canine CrCL reconstruction and to evaluate its performance in simulated surgical trials performed by both experienced and novice surgeons. We hypothesized that the aiming device would improve the accuracy and reproducibility of femoral tunnel placement across different levels of surgical experience.

## 2. Materials and Methods

### 2.1. Adjustable Aiming Device

**Figure 1:**
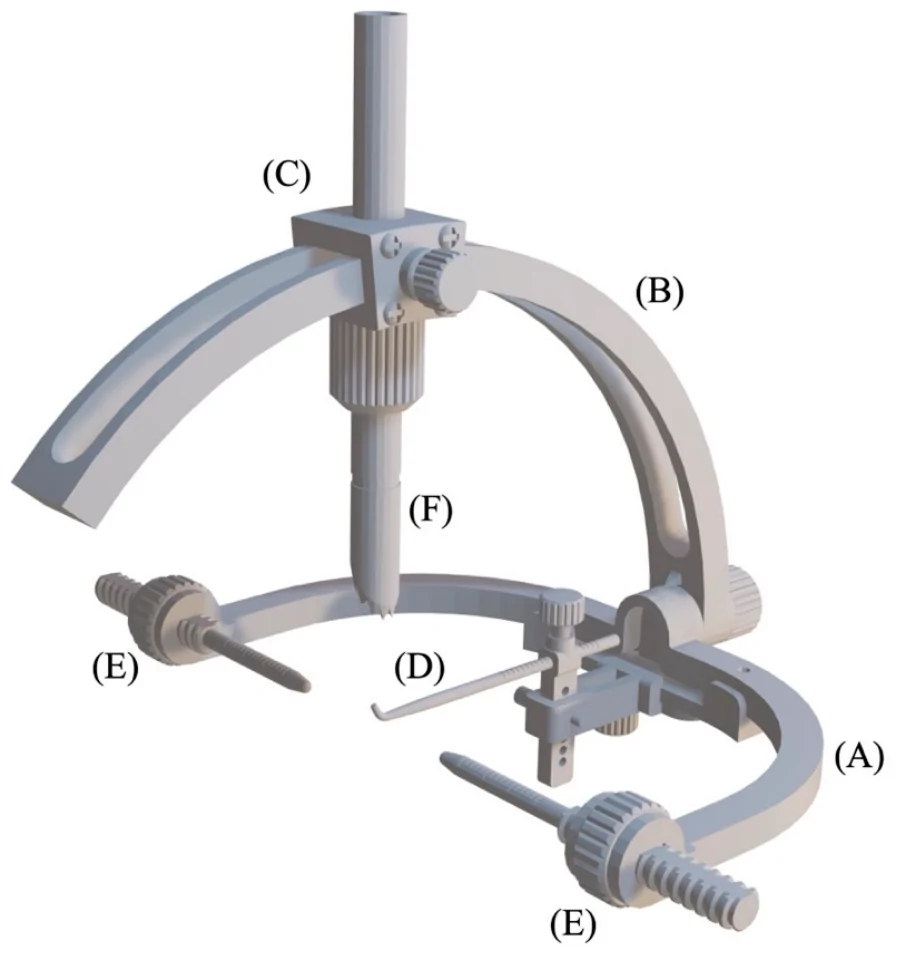
Illustration of the newly designed aiming device. The device consists of (**A**) a semicircular handle, (**B**) a one-third circular arm, and (**C**) a movable carriage, which together define the drilling angle of the bone tunnel. (**D**) An aiming hook on the handle positions the device relative to the CrCL footprint, while (**E**) bone-holding clamps secure the construct to the femur. (**F**) A drill sleeve mounted on a movable carriage is used to guide the tunnel drilling process.

The aiming device consisted of a semicircular handle (Figure 1A) connected to a one-third circular arm (Figure 1B) through an adjustable joint. A drill sleeve (Figure 1F) was mounted on a movable carriage (Figure 1C) that slides along the circular arm, allowing adjustment of the tunnel orientation. The drill sleeve was available in interchangeable sizes and contained a hollow lumen matching the diameter of the drill bit to maintain alignment during drilling. Its distal end incorporated an antislip sawtooth design, and a locking mechanism secured the sleeve to both the carriage and the arm. The handle included bone clamps (Figure 1E) to stabilize the device to the femur. An aiming hook (Figure 1D), providing three degrees of freedom, allowed fine adjustment of the device relative to the CrCL footprint. The hook, 2 mm in diameter, was sufficiently rigid to traverse the stifle joint of medium-sized dogs. Its distal tip was angled at 120° and was designed to anchor against the posterior cortex of the intercondylar notch.

### 2.2. Preoperative Planning Workflow and Device Configuration

A customized preoperative planning workflow was implemented using MATLAB (R2022b; MathWorks, Inc., Natick, MA, USA). The workflow served two purposes: (1) identifying the optimal femoral tunnel entry (FTE) point on the lateral femoral condyle (LFC) and (2) determining the device parameters to accommodate individual femoral anatomy.

**Figure 2:**
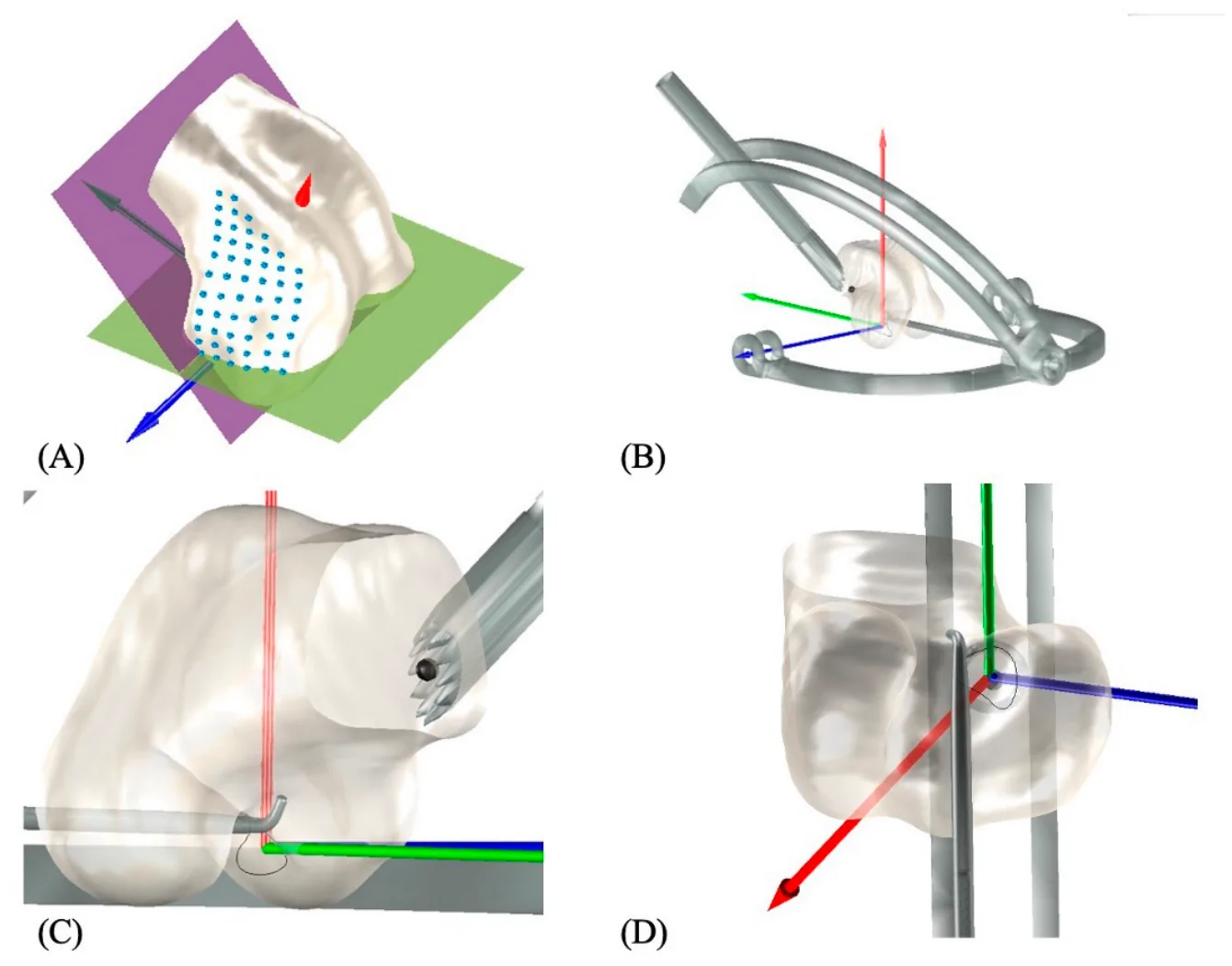
Preoperative planning process using custom-developed software based on the established orthogonal coordinate system, with the positive X-axis (red), Y-axis (green), and Z-axis (blue) directed cranially, proximally, and laterally, respectively. (**A**) Candidate femoral tunnel entry points (blue dots) were distributed across the surface of the lateral femoral condyle. (**B**) After the optimal entry point for each femur was selected, the parameters of the aiming device were iteratively adjusted within the software to best align the drill sleeve axis with the planned bone tunnel, while (**C**,**D**) the aiming hook was simultaneously positioned against the caudal cortex of the intercondylar notch.

The optimal entry point was identified using a previously established method [33]. Briefly, a CT-based 3D femoral bone model was imported, and candidate FTE points were distributed across the lateral condyle surface using a method modified from Hodel et al. [34] (Figure 2A). The CrCL footprint was estimated using a validated deformable shape template [30]. For each FTE point, a virtual cylinder (4.5 mm in diameter) was generated to simulate a femoral tunnel directed toward the centroid of the CrCL footprint. The intra-articular tunnel aperture was defined as the intersection between the simulated tunnel and the medial surface of the LFC. Three metrics were calculated: footprint similarity [33], tunnel length (distance from the intra-articular aperture centroid to the FTE points), and tunnel-face angle (angle between the tunnel axis and the surface tangent at the FTE points). The optimal entry point was defined as the candidate location satisfying the following criteria: (1) the greatest footprint similarity among the tested points, (2) a tunnel-face angle greater than 30°, and (3) a tunnel length ≥ 15 mm.

An orthogonal coordinate system was established on the femoral model to define the alignment of the aiming device (Figure 2B). The Z-axis (blue axis in Figure 2B) was aligned with the transcondylar axis of the femoral condyles. A reference plane was defined by this axis and the line connecting the third trochanter to the center of the fabella–femoral ligament. The X-axis (red axis in Figure 2B) was perpendicular to this plane, and the Y-axis (green axis in Figure 2B) was obtained from the cross product of the X- and Z-axes.

The aiming device was then virtually positioned relative to the femoral model in the planning software according to this coordinate system. The central axis of the bone-holding clamps was set parallel to the Z-axis and passed through the CrCL footprint centroid, whereas the aiming hook was oriented parallel to the Y-axis. To accommodate individual femoral anatomy, five adjustable parameters were used to configure the aiming device: the inclination angle, the arch angle, and the hook position along three orthogonal axes. The inclination angle defines the angle between the circular arm (Figure 1B) and the handle (Figure 1A), whereas the arch angle determines the position of the movable carriage along the circular arm (Figure 1C). These two parameters were adjusted to align the drill sleeve axis with the simulated femoral tunnel (Figure 2B). The hook position was then adjusted along three orthogonal axes to enable secure anchoring on the caudal cortex of the intercondylar notch (Figure 2C,D) while maintaining alignment between the drill sleeve and the planned tunnel trajectory (Figure 2B).

### 2.3. Surgical Simulation Experiment: Femoral Tunnel Positioning

#### 2.3.1. Samples

Six cadaveric hindlimbs (three left and three right) were harvested via hip disarticulation from five mixed-breed dogs (three females and two males; mean ± SD of body weight: 17.0 ± 2.2 kg) that had been euthanized for reasons unrelated to this study (IACUC number: NTU-109-EL-00070). Both hindlimbs were obtained from one dog, and one hindlimb was obtained from each of the remaining four dogs. The limbs were confirmed to be orthopedically normal or to exhibit only mild osteoarthritis on the basis of the grading system described by Marlis et al. [35]. No patient had a history of hindlimb orthopedic surgery, and physeal closure was confirmed on postmortem radiographs.

**Figure 3:**
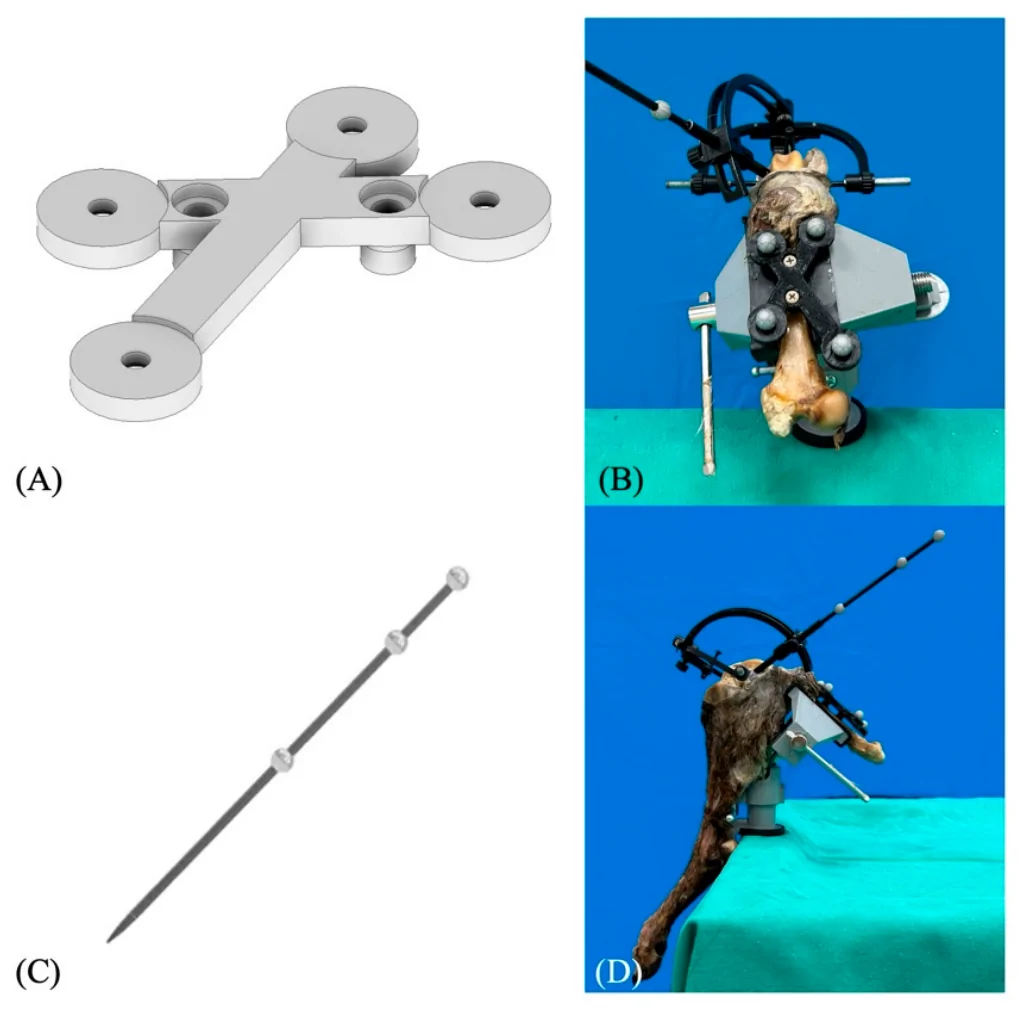
(**A**) Schematic illustration of the 3D-printed marker plate and (**B**) its placement on a cadaveric canine femur with four attached infrared reflective markers. (**C**) Photograph of the simulated drill bit embedded with three reflective markers used to define the drilling axis. (**D**) Intraoperative setup showing the application of the aiming device on a cadaveric canine hindlimb.

After being thawed at room temperature for 24 h, each limb was prepared as follows. The musculature surrounding the proximal two-thirds of the femur was removed to facilitate placement of the marker plate. The musculature surrounding the stifle joint and tibia, together with the patella and associated periarticular soft tissues, was preserved. Two holes spaced 27 mm apart were drilled into the femoral diaphysis using a 3.5 mm drill bit and tapped with a 4.0 mm tap. A custom 3D-printed marker plate (Figure 3A,B) was then fixed to the femur using two 4 mm × 30 mm polyether ether ketone (PEEK) screws and screw caps. Each limb subsequently underwent CT scanning with a voxel size of 0.636 mm × 0.636 mm × 0.300 mm. Bone segmentation was performed semiautomatically, and femoral surface meshes were reconstructed using 3D Slicer (version 4.11; Brigham and Women’s Hospital, MA, USA).

Before the experimental trials, the same investigator (TYL) prepared all limbs with a standardized midline skin incision followed by a lateral joint incision. The skin outside the incision was retained, and the patella was luxated medially during the procedure to facilitate exposure and access to the lateral aspect of the stifle. The incision provided adequate exposure for placement and manipulation of the aiming device. All surgeons used the same pre-established incision in each limb, and no appreciable enlargement of the incision was observed over successive trials.

#### 2.3.2. Participants and Testing Protocol

Thirteen veterinary surgeons participated in the study and were categorized according to surgical experience: seven novice surgical residents from the National Taiwan University Veterinary Hospital (NTUVH) with no independent experience in stifle surgery and six experienced orthopedic surgeons who had each performed more than 50 stifle procedures. The experienced group included three board-certified small-animal surgeons (DACVS-SA) and three senior orthopedic veterinarians practicing in Taiwan. None of the participants had prior experience using the new aiming device. Experimental sessions were conducted at the NTUVH between August 2023 and April 2024.

Before testing, all participants attended a standardized training session consisting of a lecture on canine stifle anatomy and instructions for using the aiming device. This was followed by hands-on practice with both the aiming device and the freehand technique, first on a synthetic femoral model and then on a cadaveric hindlimb excluded from the formal trials. During the formal trials, each surgeon performed femoral tunnel placement on each limb using both the aiming device and the freehand technique. For each technique, the time required for instrument setup (aiming device or goniometer and calipers) was recorded, followed by the time required to perform simulated tunnel placement. The order of technique and limb assignment was randomized to minimize potential order effects. One of the authors (TYL) served as a surgical assistant in all the procedures to maintain procedural consistency.

#### 2.3.3. Motion Capture System

The orientation and position of the femur and simulated bone tunnel were recorded using a motion capture (MoCap) system consisting of seven infrared-reflective cameras (four Bonita 10 and three Vero v2.2, VICON, Oxford Metrics, Oxford, UK). The cameras were arranged in a circular configuration approximately 1.5 m from the surgical table. A custom clamping device was mounted at the edge of the table to secure the cadaveric hindlimb in a position that simulated typical stifle surgeries. The MoCap system acquired data at a sampling rate of 120 Hz and was calibrated in accordance with the manufacturer’s protocols. Reflective marker clusters were attached to both the drill bit and the femoral marker plate (Figure 3A,B). During each trial, the spatial positions of these markers were recorded for three seconds for each tunnel positioning attempt.

#### 2.3.4. Femoral Tunnel Positioning Using the Aiming Device

For the aiming device technique, the device was first configured according to the parameters obtained from the preoperative planning workflow. The aiming hook traversed the stifle joint and was anchored against the caudal cortex of the intercondylar notch. The device was then secured to the femur using two bone-holding clamps while maintaining the clamp axis parallel to the transcondylar axis of the femoral condyles. After fixation, the drill sleeve was positioned close to the lateral femoral cortex, and a simulated drill bit was inserted through the sleeve to represent the planned tunnel trajectory (Figure 3C,D). Once the surgeon confirmed correct positioning, the markers on the femoral marker plate and drill bit were recorded using the MoCap system.

#### 2.3.5. Freehand Femoral Tunnel Positioning

The optimal femoral tunnel entry point and tunnel trajectory were obtained from the preoperative planning workflow described above. A custom MATLAB script calculated the distances from the planned entry point to two anatomical reference landmarks: the center of the best-fit circle of the LFC and the midpoint of the fabella–femoral ligament (FFL). The planned tunnel orientations in the sagittal and coronal planes were also computed. These measurements were provided to the surgeons to guide freehand positioning.

During the trials, participants first identified the FFL and the LFC centers by visual inspection and palpation. Calipers were used to reproduce the preoperatively determined distances from these landmarks to the femoral entry point. Two arcs were then drawn from the FFL and LFC centers using these distances as radii, and their intersection defined the intended entry point on the lateral femoral condyle. The drill bit was positioned at the femoral entry point and aligned according to the planned tunnel orientation using a goniometer and visual guidance. The drill bit was then inserted through a universal drill guide, and the marker positions on the drill bit and femoral plate were recorded using the MoCap system.

### 2.4. Tunnel Trajectory Simulation

Following data acquisition, the 3D coordinates of each reflective marker were reconstructed and labeled using Nexus software 2.15 (VICON). To integrate the MoCap data with the CT-based femoral mesh model, spatial registration was performed between the CT coordinate system (CTS) and the MoCap coordinate system (MCS). Using the markers attached to the femur, a rigid transformation between the CTS and MCS was computed, allowing the CT-based femoral mesh model to be expressed within the MCS.

For each tunnel positioning task, the coordinates of the reflective markers attached to the drill bit were extracted from the MoCap recordings. Marker trajectories acquired during the recording period were temporally averaged to obtain representative marker positions. A least-squares best-fit line through the averaged marker positions defined the drill bit axis, representing the trajectory of the drilled tunnel. The center of the intra-articular aperture of the tunnel was determined by calculating the intersection between the drill bit axis and the medial surface of the lateral femoral condyle on the reconstructed femoral model.

### 2.5. Preliminary Feasibility Assessment of Actual Device-Guided Femoral Tunnel Drilling

After all 13 surgeons completed the simulated positioning trials, actual device-guided femoral tunnel drilling was performed on the same six cadaveric hindlimbs to assess the practical feasibility of the complete drilling procedure. One experienced orthopedic surgeon operated on three limbs, and one novice surgeon operated on the remaining three limbs, with one tunnel drilled per limb. For each limb, the aiming device was configured according to the preoperative planning parameters. A 1.4 mm K-wire was first inserted through the drill sleeve to establish the tunnel trajectory, after which a guided hole was drilled. The device was then detached, and a 2.5 mm cannulated drill bit was used to create the bone tunnel, which was subsequently enlarged to a final diameter of 4 mm. Postdrilling CT scans were obtained for all limbs, and the femoral surface meshes were reconstructed using 3D Slicer for quantitative evaluation of tunnel accuracy.

### 2.6. Error Metrics

Positional deviations between the simulated intra-articular tunnel aperture center and the centroid of the femoral CrCL footprint (i.e., the planned target location) were quantified along the cranial–caudal, proximal–distal, and mediolateral axes. These axis-specific deviations were defined as component errors, where deviations from zero indicated systematic bias in tunnel placement along the corresponding direction. The Euclidean distance (ED) between the simulated tunnel aperture center and the predicted CrCL footprint centroid was calculated to represent the overall tunnel aperture error. In addition, the angular deviation (AD) between the planned tunnel axis and the simulated drill-bit axis was calculated to evaluate the directional accuracy. To quantify the occurrence of extreme tunnel placement errors, placements in which the simulated tunnel trajectory exited outside the intended intra-articular region on the medial surface of the lateral femoral condyle, such as through the distal femoral cortex or medial femoral condyle, were operationally classified as failures. For each technique, the failure rate was calculated by dividing the number of failed positioning trials by the total number of positioning trials and multiplying by 100%.

### 2.7. Statistical Analysis

Descriptive statistics (means ± SDs) were calculated for component errors, ED, AD, and positioning durations. For ED, AD, and positioning duration, measurements obtained from the six limbs were averaged for each surgeon and technique before statistical analysis. The effects of surgical technique (aiming device vs. freehand), surgeon experience (experienced vs. novice), and their interaction were evaluated using two-way mixed ANOVA, with technique as a within-subject factor and experience as a between-subject factor. When a significant interaction was detected, pairwise comparisons were performed with a Bonferroni adjustment. Normality was assessed using the Shapiro–Wilk test. Systematic directional bias in tunnel aperture placement was evaluated separately for each coordinate axis using one-sample t tests or Wilcoxon signed-rank tests, as appropriate.

Intrasurgeon variability across limbs was quantified as the standard deviation of the ED and AD across the six limbs for each surgeon and technique. These dispersion measures were analyzed using two-way mixed ANOVA with technique as the within-subject factor and experience as the between-subject factor. Intersurgeon variability was quantified for each limb as the standard deviation of the ED and AD across the six surgeons within each experience group. Intersurgeon variability was compared between the aiming device and freehand techniques using paired t tests or Wilcoxon signed-rank tests, as appropriate. All the statistical analyses were performed using MATLAB, and statistical significance was set at *p* < 0.05.

## 3. Results

### 3.1. Femoral Tunnel Positioning Performance

**Figure 4:**
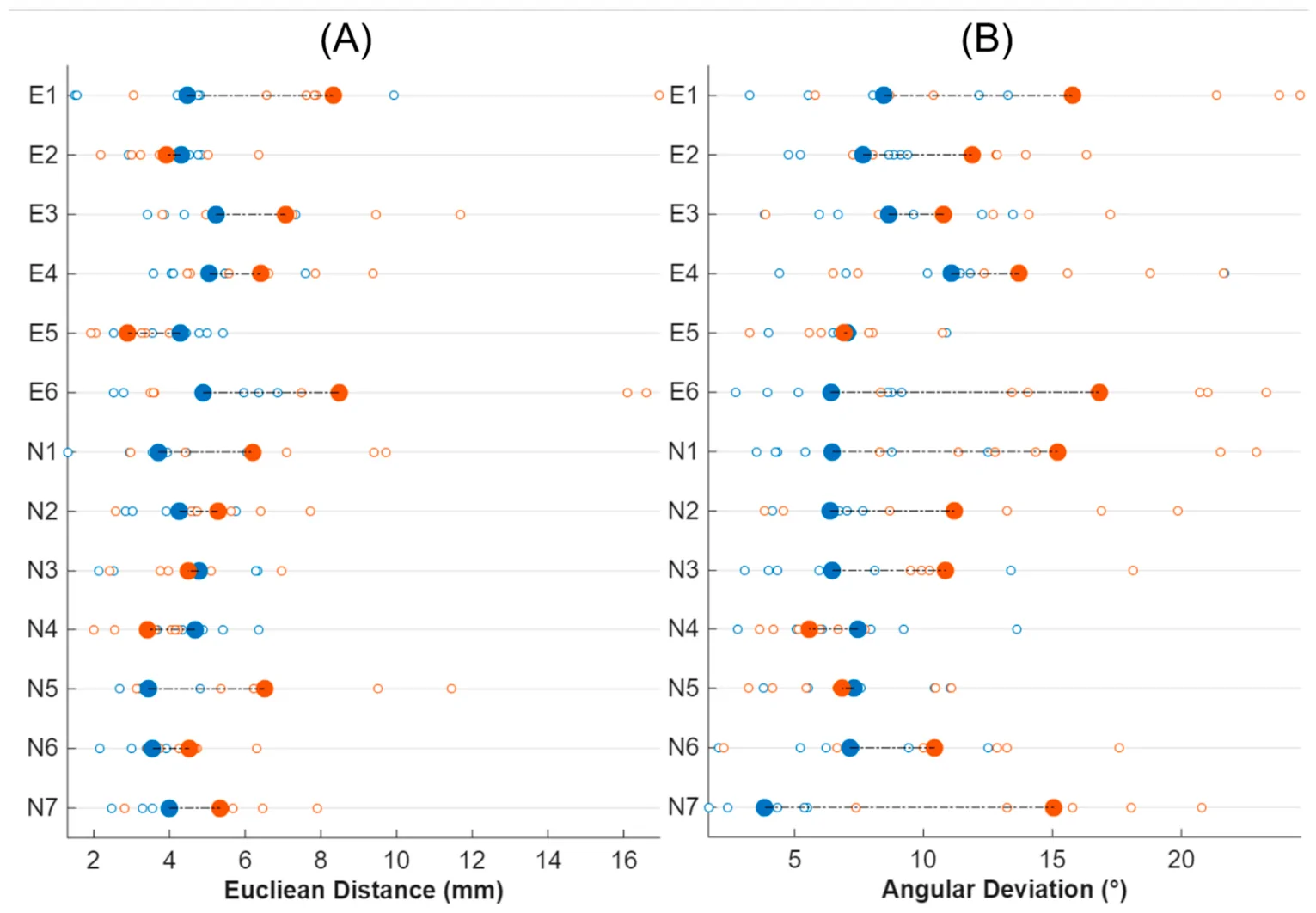
Femoral tunnel positioning errors using the aiming device and freehand techniques: (**A**) tunnel aperture error quantified by Euclidean distance and (**B**) angular deviation. Hollow circles represent individual cadaveric limbs, whereas filled circles represent the mean across six limbs for each surgeon. The dashed lines connect the mean values obtained with the two techniques. E1–E6 and N1–N7 denote experienced and novice surgeons, respectively. Blue and orange denote the aiming device and freehand techniques, respectively.

The femoral tunnel positioning errors for each experimental limb, stratified by surgeon and surgical technique, are shown in Figure 4. As summarized in Table 1, compared with the freehand technique, the use of the aiming device resulted in lower tunnel aperture error (ED) for both experienced surgeons (4.7 ± 0.4 vs. 6.2 ± 2.3 mm) and novice surgeons (4.1 ± 0.5 vs. 5.1 ± 1.1 mm). The main effect of surgical technique on ED was significant (*p* = 0.0275), whereas neither the main effect of surgeon experience nor the interaction effect was significant. Similarly, compared with the freehand technique, the aiming device produced a smaller AD in both the experienced (8.2 ± 1.6° vs. 12.6 ± 3.6°) and novice (6.4 ± 1.2° vs. 10.7 ± 3.7°) groups. A significant main effect of surgical technique was observed for AD (*p* = 0.0038). However, neither surgeon experience nor their interaction had a significant effect on AD.

When the data from both experience groups were pooled, the failure rate decreased from 13.8% with the freehand technique to 0.4% with the aiming device. The aiming device also significantly reduced the tunnel positioning time in both experience groups (*p* = 0.0005), with mean reductions of 45.2 s for experienced surgeons and 73.0 s for novice surgeons (Table 1).

### 3.2. Component Errors

**Figure 5:**
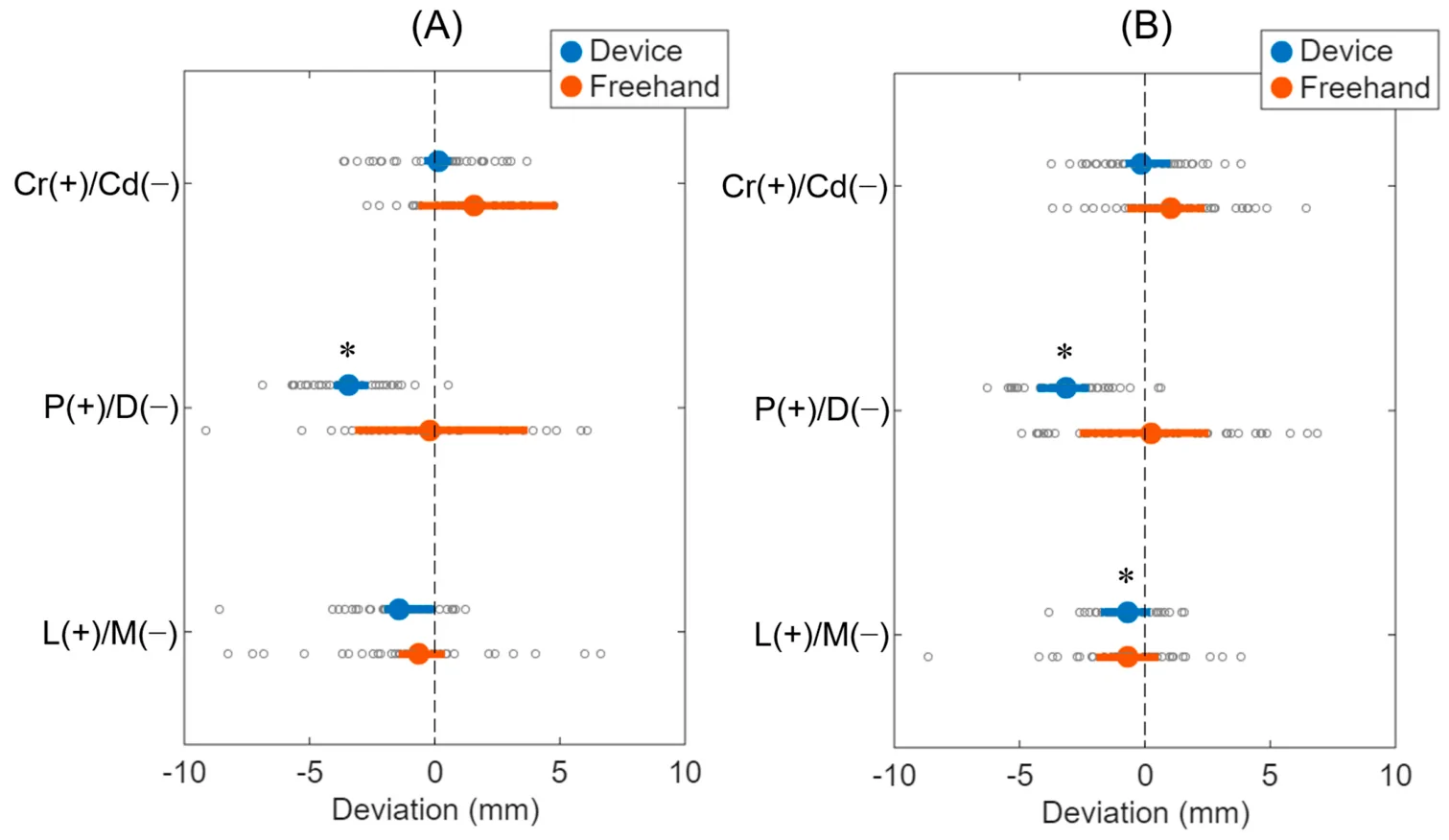
Directional aperture errors for the aiming device and freehand techniques for (**A**) experienced surgeons and (**B**) novice surgeons. Hollow circles represent component errors for individual cadaveric limbs, whereas filled circles indicate the mean component error across surgeons. The horizontal line segments indicate the range of surgeon-level mean values. Blue and orange denote the aiming device and freehand techniques, respectively. Positive and negative values indicate opposite directions along each coordinate axis (Cr/Cd, cranial/caudal; P/D, proximal/distal; L/M, lateral/medial). An asterisk indicates a mean value significantly different from zero (*p* < 0.05).

The signed component errors for the two surgical techniques are presented in Figure 5. In the cranial–caudal direction, the mean deviations obtained using the aiming device were closer to zero than those obtained using the freehand technique for both experienced (0.1 ± 0.4 vs. 1.6 ± 1.9 mm) and novice surgeons (−0.2 ± 0.5 vs. 1.0 ± 1.2 mm), although no significant systematic bias was detected in either experience group. In the proximal–distal direction, the aiming device produced mean distal deviations of 3.4 ± 0.5 mm for experienced surgeons and 3.1 ± 0.6 mm for novice surgeons, both of which represented significant biases from the target position (*p* < 0.001). In the medial–lateral direction, the mean deviations were generally negative, indicating a tendency toward medial placement; however, a significant medial bias was identified only for the aiming device among novice surgeons (*p* = 0.0208), whereas the remaining conditions showed no significant bias. Overall, component errors obtained using the aiming device appeared less dispersed than those obtained using the freehand technique, particularly in the cranial–caudal and proximal–distal directions.

### 3.3. Intra- and Intersurgeon Variability

The aiming device reduced the intrasurgeon variability in tunnel positioning (Table 2). The mean intrasurgeon SD of the ED decreased from 3.0 ± 2.1 to 1.6 ± 0.8 mm for experienced surgeons and from 1.9 ± 0.9 to 1.3 ± 0.5 mm for novice surgeons, with a significant technique effect (*p* = 0.0189). Similarly, the mean intrasurgeon SD of the AD decreased from 5.2 ± 2.1° to 3.4 ± 1.4° and from 4.5 ± 1.7° to 2.9 ± 1.1° in the experienced and novice groups, respectively (*p* = 0.0181). Neither the experience effect nor the interaction was significant for either outcome (all *p* > 0.05).

**Figure 6:**
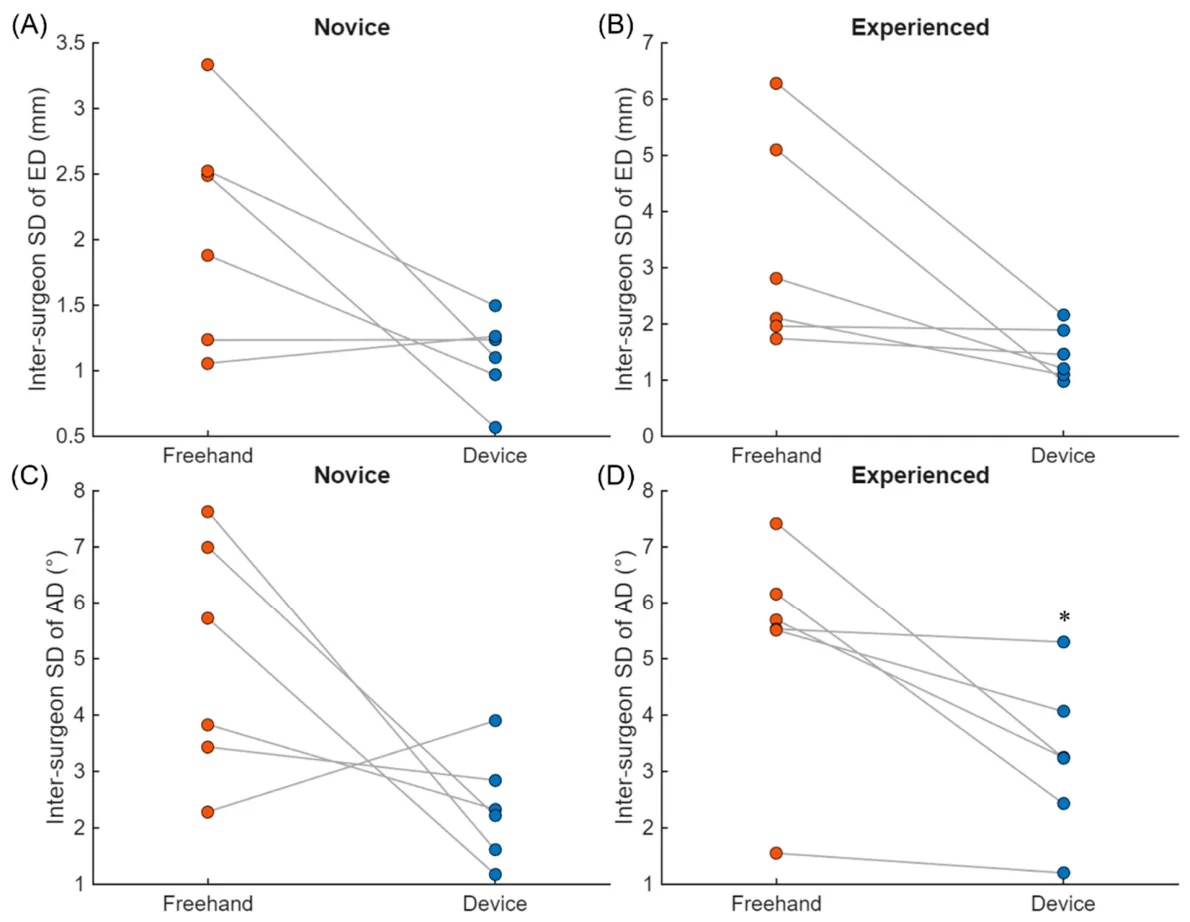
Intersurgeon variability in femoral tunnel positioning using freehand and aiming device techniques. Intersurgeon variability was quantified as the standard deviation (SD) across surgeons for each cadaveric limb. Panels show the Euclidean distance (ED) for (**A**) novice and (**B**) experienced surgeons and the angular deviation (AD) for (**C**) novice and (**D**) experienced surgeons. The connected points represent the same limb under the two techniques. Orange and blue indicate the freehand and aiming device techniques, respectively. * indicates a significant difference between techniques (*p* < 0.05).

The mean intersurgeon SDs of the ED and AD were lower with the aiming device than with the freehand technique in both experience groups (Figure 6). However, the reduction was statistically significant only for AD in the experienced group (*p* = 0.0298; Figure 6D). No significant differences were observed for ED in either group (Figure 6A,B) or for AD in the novice group (Figure 6C).

### 3.4. Device-Guided Femoral Tunnel Drilling in Cadaveric Limbs

During actual femoral tunnel drilling, the intra-articular apertures of the final four tunnels were visually located within the CrCL footprint, with postoperative aperture errors ranging from 1.8 to 4.0 mm (Table 3). Notably, during the second drilling procedure, the tunnel aperture was positioned at the cartilage margin of the lateral femoral condyle during the simulated positioning stage but penetrated the articular cartilage during actual drilling. Following this incident, the drilling protocol was modified by introducing an intermediate 3.5 mm pin between the 2.5 mm drill bit and the final 4.0 mm drill bit to reduce the risk of thread stripping. Overall, the drilled tunnels exhibited distal displacement, with a mean proximal–distal component error of −3.7 ± 3.0 mm, which is consistent with the distal bias observed in the simulated positioning trials.

## 4. Discussion

The results of the present study demonstrated that the aiming device improved the accuracy, reproducibility, and procedural efficiency of femoral tunnel placement. Regardless of surgeon experience, compared with the freehand technique, the use of the device significantly reduced the intra-articular aperture positional error, angular deviation, and positioning time. Lower errors were consistently observed among both experienced and novice surgeons, suggesting that the benefits of the device were not restricted to a particular level of surgical experience. The device also reduced intrasurgeon variability, indicating improved repeatability among individual surgeons. Nevertheless, component-wise analysis revealed systematic distal and medial deviations, indicating that these improvements were accompanied by consistent directional biases likely attributable to the current device design and its surgical application.

The design principles of the present aiming device were adapted from instruments previously described for human orthopedic surgery [36,37,38,39,40], with modifications to accommodate the anatomy and surgical requirements of the canine stifle. Integration with custom-developed planning software allowed the planned aperture location and tunnel trajectory to be converted into preoperatively defined device settings, thereby providing a standardized means of transferring patient-specific surgical plans to the surgical setting. Consistent with this design objective, the use of the aiming device significantly reduced both ED and AD across surgeons’ experience levels (Table 1). Improvements were observed in both groups, suggesting that the accuracy benefits of the device were not strongly dependent on prior surgical experience. The reduction in AD is particularly relevant because previous human studies have shown that excessive vertical tunnels may compromise rotational stability, whereas overly oblique tunnels may increase the risk of posterior wall blowout [16,41]. Although these findings cannot be directly extrapolated to the canine stifle, they underscore the potential clinical importance of accurately controlling the tunnel trajectory. The observed reductions in intra- and intersurgeon variability in the ED and AD (Table 2 and Figure 6) may likewise reflect the standardized, preoperatively determined device configuration, which reduces reliance on subjective intraoperative estimation and promotes more reproducible tunnel placement. Although the well-configured aiming device still required intraoperative alignment with palpable or visually identifiable anatomical landmarks, all surgeons were able to complete the procedure after a single practice session. This provides preliminary evidence that the device can be adopted with limited initial training; however, because performance was not evaluated across multiple training sessions, its learning curve could not be formally assessed.

Component-wise analysis showed lower intersurgeon dispersion with the aiming device, together with improved control of the cranial–caudal component of the placement error; however, the device-guided tunnels were systematically displaced distally and medially relative to the planned target (Figure 5). The absence of systematic cranial displacement of the intra-articular aperture may be favorable because it can reduce the risk of excessive cranial tunnel placement. Although anatomical and biomechanical implications are not equivalent between the human knee and canine stifle, excessive anterior femoral tunnel placement in humans has been associated with restricted knee flexion, increased graft tension, and potentially compromised postoperative function [42]. This comparison provides only an indirect clinical context, and whether preventing cranial displacement offers similar benefits in dogs remains to be determined. Conversely, distal and medial displacement can alter graft orientation, but its biomechanical and clinical consequences in the canine stifle remain unexplored.

The observed directional biases in intra-articular aperture placement may reflect the combined effects of device design, operator–device interaction, and limb-specific anatomy. The current aiming hook permits vertical adjustment only in 2 mm increments, which may limit its ability to reproduce the planned position. Moreover, the hook tip has a fixed angle of 120°, which is derived from preliminary anatomical measurements in a small, separate sample of dogs. This fixed geometry may not conform equally well to individual variations in the relationship between the intercondylar roof and the adjacent caudal femoral cortex, potentially contributing to proximal–distal positioning errors. A distal shift may also be introduced when the surgeon applies distal traction to obtain clearer tactile confirmation that the hook has engaged the caudal aspect of the intercondylar roof. In addition, the geometry of the bone-holding clamp may encourage placement near the central portion rather than the intended caudal margin of the femoral condyle, thereby altering the orientation of the guide. This issue may be further exacerbated by the surrounding soft tissue in cadaveric limbs, which can obscure relevant bony landmarks and prevent consistent seating of the clamp.

Several design modifications could be considered to address these limitations. The current stepwise vertical adjustment could be replaced with a lockable sliding-rail mechanism, allowing continuous adjustment of the aiming-hook position. The hook geometry could also be refined to provide more secure engagement with the caudal cortex of the femoral condyle or the caudal wall of the lateral femoral condyle, thereby reducing the need for additional distal traction. Alternatively, patient-specific aiming hooks could be fabricated using 3D printing technology, with their geometry, offset, and angulation determined from preoperative imaging and surgical planning. The bone-holding clamp could also be redesigned to register at the more intuitive central region of the femoral condyle, with the guide geometry recalibrated accordingly to preserve the tunnel trajectory. To reduce the influence of soft-tissue interference, the device could incorporate additional visual alignment features referenced to more readily exposed anatomical landmarks, such as the trochlear ridge. Collectively, these modifications may reduce the observed directional biases while preserving the improved consistency provided by the aiming device.

Beyond improving positioning accuracy, the near elimination of extreme tunnel placement errors with the aiming device suggests that it may provide an additional safeguard against gross malposition and potentially reduce the risk of iatrogenic cartilage damage or cortical blowout. However, because the present simulation-based assessment did not evaluate actual surgical complications, larger cadaveric drilling and clinical studies are needed to determine whether this reduction translates to fewer intraoperative complications. Device use also reduced the intraoperative tunnel positioning time (Table 1). This measurement excluded preoperative planning time and therefore primarily reflected the shorter time required for device adjustment and positioning than for repeated visual alignment of the drill bit with the anatomical landmarks during freehand placement.

Although none of the differences in the experience group reached statistical significance, novice surgeons showed slightly lower mean ED and AD and lower mean intrasurgeon variability in both outcomes under both freehand and device-guided conditions (Table 1 and Table 2). These numerical differences should not be interpreted as evidence that novice surgeons performed better, as they may reflect sampling variation. One possible explanation for the comparable performance between the two groups is that despite the experienced surgeons’ greater general anatomical and orthopedic knowledge, both groups had limited prior experience with anatomic ICR, and none of the participants had been involved in the development of the aiming device. In the device-guided condition, all device settings were derived directly from the patient-specific surgical plan, creating a standardized workflow that may have further reduced the reliance on experience-dependent spatial judgment. This multioperator experimental design also extends the findings of Bolia et al., who reported favorable outcomes using an arthroscopically assisted femoral tunnel aiming guide but evaluated the device with a single experienced operator familiar with its use [32]. By including multiple operators with varying levels of surgical experience, the present study captured operator-related variability and provided a more representative indication of how the device might perform when used by a broader range of surgeons.

Several limitations should be acknowledged. First, the primary experiment evaluated drill bit positioning rather than the accuracy of the completed bone tunnels. Although actual drilling was performed in six cadaveric limbs, this component served only as a preliminary feasibility assessment and was insufficient to draw definitive conclusions about drilling accuracy. Second, the proposed workflow requires preoperative CT imaging, which, although increasingly available in veterinary medicine, is not yet routinely accessible in general practice and entails additional cost. Moreover, the limited sample of six hindlimbs from five dogs may not adequately represent the anatomical variation in the broader canine population. The specimens were obtained from dogs without or with only mild osteoarthritis; therefore, the applicability of the device to dogs with advanced osteoarthritis, substantial osteophyte formation, or skeletal deformities remains uncertain. In addition, progressive soft-tissue laxity was observed with repeated specimen use, potentially facilitating hook placement in later trials. These tissue changes and their effects on positioning performance were not systematically assessed. Finally, all surgeons received only a single practice session, and performance was not evaluated longitudinally. The learning curve and the influence of additional practice on accuracy and procedural efficiency could not be determined.

## 5. Conclusions

This is the first study to evaluate a patient-specific, planning-guided approach to femoral tunnel placement in the canine stifle. Compared with the freehand technique, the use of the custom-aiming device and planning software significantly improved the accuracy of intra-articular aperture positioning and tunnel orientation and reduced intrasurgeon variability. These benefits were observed for both experienced and novice surgeons, and the absence of a significant effect of surgical experience suggests that performance in this specific procedure may be less dependent on orthopedic experience. Nevertheless, systematic distal and medial biases were identified, likely reflecting limitations in the current device geometry and operator–device interaction. Although these biases may be addressable through further device refinement and continued hands-on training, additional cadaveric and clinical validation is needed to determine whether the observed improvements translate into more reliable tunnel creation and better postoperative outcomes.

## Figures and Tables

**Table 1:** Comparison of femoral tunnel positioning accuracy and positioning time between surgical techniques and surgeon experience groups. The data are presented as the mean ± standard deviation. *p* values were obtained from a two-way mixed ANOVA with surgical technique as the within-subject factor and surgeon experience as the between-subject factor. ED, Euclidean distance; AD, angular deviation.

Outcome	Experienced Freehand	Experienced Device	Novice Freehand	Novice Device	Technique Effect, *p*	Experience Effect, *p*	Technique × Experience, *p*
ED (mm)	6.2 ± 2.3	4.7 ± 0.4	5.1 ± 1.1	4.1 ± 0.5	0.0275	0.1186	0.6729
AD (°)	12.6 ± 3.6	8.2 ± 1.6	10.7 ± 3.7	6.4 ± 1.2	0.0038	0.0839	0.9534
Positioning time (s)	200.9 ± 54.7	155.7 ± 38.1	230.8 ± 80.3	157.8 ± 41.0	0.0005	0.5944	0.2750

**Table 2:** Intrasurgeon variability in femoral tunnel positioning using the freehand and aiming device techniques. Variability was quantified as the standard deviation of the ED and AD across the six cadaveric limbs for each surgeon. The values are presented as the mean ± standard deviation. *p* values were obtained from a two-way mixed ANOVA with surgical technique as the within-subject factor and surgeon experience as the between-subject factor. ED, Euclidean distance; AD, angular deviation.

Outcome	Experienced Freehand	Experienced Device	Novice Freehand	Novice Device	Technique Effect, *p*	Experience Effect, *p*	Technique × Experience, *p*
ED (mm)	3.0 ± 2.1	1.6 ± 0.8	1.9 ± 0.9	1.3 ± 0.5	0.0189	0.2056	0.3560
AD (°)	5.2 ± 2.1	3.4 ± 1.4	4.5 ± 1.7	2.9 ± 1.1	0.0181	0.3883	0.8935

**Table 3:** Component and aperture errors following actual device-guided femoral tunnel drilling in six cadaveric limbs. The first three limbs were operated on by an experienced surgeon and the remaining three by a novice surgeon. Limbs are listed chronologically according to the drilling sequence. Cr/Cd, cranial/caudal; P/D, proximal/distal; L/M, lateral/medial; ED, Euclidean distance.

	Limb Sequence		Component Error (mm)			Tunnel Aperture Error (mm)
			Cr(+)/Cd(−)	P(+)/D(−)	L(+)/M(−)	ED
Experienced surgeon	Earlier 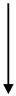 Later	1	2.8	−4.5	−1.7	5.6
		2	−1.6	−9.2	−2.9	9.7
		3	−2.6	−2.0	−1.1	3.5
Novice surgeon		4	0.8	−3.8	−0.7	4.0
		5	0.3	−2.2	−0.2	2.3
		6	1.6	−0.4	0.7	1.8
Total			0.2 ± 2.0	−3.7 ± 3.0	−1.0 ± 1.2	4.5 ± 2.9

## Data Availability

The data supporting the findings of this study are available upon reasonable request from the corresponding author.
